# Maternal human habituation enhances sons’ risk of human-caused mortality in a large carnivore, brown bears

**DOI:** 10.1038/s41598-020-73057-5

**Published:** 2020-10-05

**Authors:** Michito Shimozuru, Yuri Shirane, Masami Yamanaka, Masanao Nakanishi, Tsuyoshi Ishinazaka, Shinsuke Kasai, Takane Nose, Masataka Shirayanagi, Mina Jimbo, Hifumi Tsuruga, Tsutomu Mano, Toshio Tsubota

**Affiliations:** 1 Laboratory of Wildlife Biology and Medicine, Faculty of Veterinary Medicine, Hokkaido University, Kita 18 Nishi 9, Kita-ku, Sapporo, Hokkaido 060-0818 Japan; 2 Shiretoko Nature Foundation, 531 Iwaubetsu, Shari, Hokkaido 099-4356 Japan; 3 Hokkaido Research Organization, Kita 19 Nishi 11, Kita-ku, Sapporo, Hokkaido 060-0819 Japan

**Keywords:** Ecology, Behavioural ecology, Conservation biology, Molecular ecology

## Abstract

Human habituation of large carnivores is becoming a serious problem that generates human–wildlife conflict, which often results in the removal of animals as nuisances. Although never tested, human habituation potentially reduces the fitness of adult females by reducing their offspring’s survival as well as their own, due to an increased likelihood of human-caused mortality. Here, we tested this hypothesis in brown bears inhabiting Shiretoko National Park, Japan. We estimated the frequency of human-caused mortality of independent young (aged 1–4 years) born to mothers living in areas with different maternal levels of human habituation and different proximities to areas of human activity. The overall mortality rate was higher in males than in females, and in females living near a town than those in a remote area of park. Surprisingly, more than 70% of males born to highly habituated mothers living around a remote wildlife protection area were killed by humans; this proportion is greater than that for males born to less-habituated mothers living in almost the same area. The current study clarified that interactions among maternal human habituation, birthplace (proximity to town), age, and sex determine the likelihood of human-caused mortality of brown bears at an early stage of life.

## Introduction

Habituation is a process leading to a decrease in response to a repeated stimulus, and is often adaptive in that it decreases the likelihood that individuals will respond to harmless stimuli^1^. Some wild animals become habituated to humans, which is defined as the waning of an animal’s flight response to humans following repeated exposure to human presence and activities^2–4^. Human habituation has been reported for a variety of vertebrates, including mammals^5–7^, birds^8^, reptiles^9^, and fishes^10^. This phenomenon, likely to occur in populated areas, such as busy tourist wildlife sanctuaries, can provide valuable experiences of nature to tourists and economic benefits to local communities^11^. However, it can have negative impacts on wildlife conservation and management by enhancing the risk of infectious disease transmission from humans^12^, increasing the vulnerability of prey animals to predators^13^, and enhancing the risk of traffic accidents^14^. In addition, the conditioning of habituated animals, such as large carnivores, to anthropogenic foods increases the risks of crop damage and attacks on humans, domestic animals, and livestock^15^.

Brown bears (*Ursus arctos*) are among the largest carnivores and are highly adapted to the Northern Hemisphere. Human habituation has been reported in several bear habitats in North America, including bear-viewing areas throughout Alaska, where bears are highly aggregated due to the occurrence of salmon running^16^, and Yellowstone National Park, which a huge number of tourists visit^3^. This has also been reported in European brown bears^17^, albeit for fewer cases than for bears in North America, probably due to long-term persecution until the late twentieth century or higher hunting pressure^18,19^. Although human habituation could benefit bears by increasing their access to high-quality natural foods and other resources adjacent to roads, developments, and bear-viewing areas^3,4,16^, it has been considered to place bears at greater risk of human-caused mortality, including by management killing, hunting, poaching, and traffic accidents^3,20–22^. Indeed, habituated individuals were more likely to become food conditioned, and were killed by humans approximately threefold as often as were non-habituated individuals, during the 1970s and 1980s in Yellowstone National Park^21^. The likelihood of a bear becoming habituated to humans, and thus sometimes being removed as a nuisance, depends on multiple factors, such as sex, age, bear density, visitor density, and proximity of the home range to areas of human activity (e.g., towns and roads)^16,21,22^. In addition, it is empirically supposed that the offspring of habituated mothers become tolerant of humans^23^. Thus, human habituation conceivably reduces the fitness of adult females by reducing not only their own survival, but also that of their offspring, due to an increased likelihood of human-caused mortality. To our knowledge, however, this hypothesis has not been verified.

Many studies of laboratory and captive animals have suggested that the parental human habituation level is inherited by the offspring, through genetic and environmental factors, and their interactions^24–26^. However, it is not known how the parental habituation affects the offspring’s survival and future reproductive success in wildlife or laboratory/captive conditions. In wildlife inhabiting near areas of human activity, survival depends largely on human-caused death (i.e., management killing, hunting, traffic accidents, etc.)^27^. In general, the likelihood of human killing is dependent on the species and its conservation status (e.g., endangered or not), and its different impacts on human activity (e.g., potential risk of crop damage and attacks on humans, domestic animals, and livestock). The occurrence of human killing is also dependent on the habitat (e.g., protected or unprotected areas, and distance between the habitat and areas of human activity). Thus, the influence of parental human habituation on the survival of offspring should be tested for various species in various populations. However, this approach needs long-term continuous surveys, which make it difficult to address this issue.

**Figure 1:**
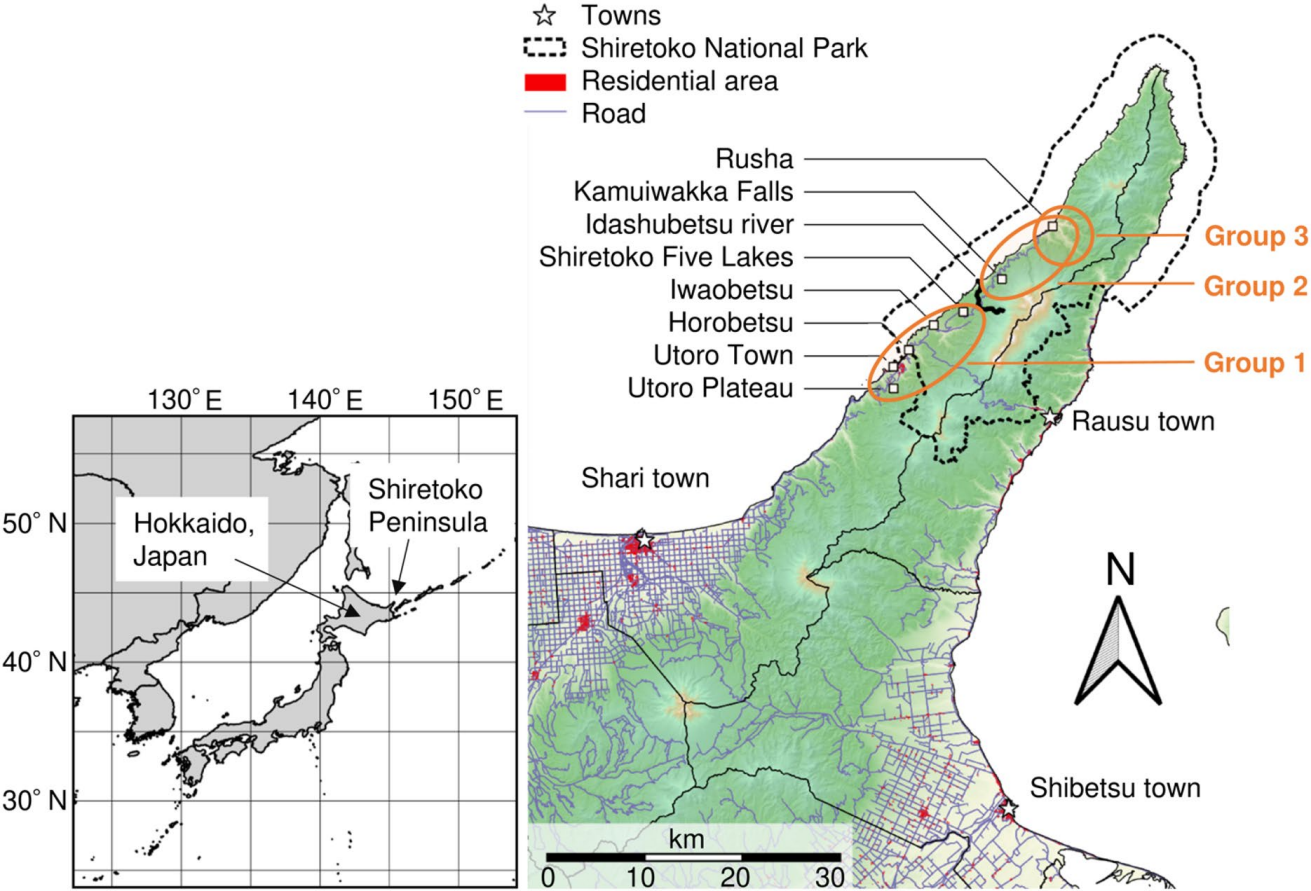
Map of the Shiretoko Peninsula, eastern Hokkaido, Japan. The dotted black line indicates the Shiretoko National Park. Orange circles indicate the area which include the mother bears in each group. This map was created using QGIS version 2.16 (QGIS Development Team, 2017. QGIS Geographic Information System. *Open Source* Geospatial Foundation Project. https://qgis.osgeo.org) and edited by M. Shimozuru. The base-map image, contour lines, topographic features are based on the National Land Numerical Information published by National Spatial Planning and Regional Policy Bureau, Ministry of Land, Infrastructure, Transport, and Tourism of Japan (available from , accessed 7 December 2017).

The Shiretoko Peninsula is located in eastern Hokkaido, Japan (Fig. 1). An area extending from the middle to the tip of the peninsula has been designated a UNESCO World Natural Heritage Site because of its remarkable ecosystems and biodiversity, and as a national park where bear habitat is protected. However, human–bear conflict, including agricultural crop depredation and intrusion into human residential areas, is a problem on the peninsula. As many as 20–70 bears have been killed annually over the past decade, mainly for management purposes^28^. In addition, the emergence of human-habituated bears, especially in sightseeing areas in the front portion of the park (here, defined as the peripheral area close to human residences), has become a serious problem for park management. Habituation increased the number of bear sightings in the town of Shari from < 100 in 1994 to > 500 in 1998^28^ and > 1000 in 2012 (our unpublished data), which has increased the risk of bear-related accidents for residents and tourists. At the same time, however, bears have become one of the most attractive tourism resources on the peninsula^29^. Many tourists visit the coastal area of the backcountry portion of the national park by yacht to see wild bears that are highly habituated to humans (Fig. S1). Those bears had been considered unrelated to conflict with humans because they live in a remote area of the national park, but recently it came to light that the bears born in this area sometimes intrude into human residential areas and are killed as nuisances. For proper conservation and management of bears, the effect of habituation on the occurrence of human–bear conflict and the population dynamics of bears need to be evaluated.

In this study, to clarify how multiple factors, including human habituation of the mother, sex, age, and proximity of the birthplace to areas of human activity, affect the likelihood of human-caused death of offspring, we tested the following five hypotheses: (1) male offspring are more likely to be killed than are females, due to male-biased natal dispersal^30^ (namely, “sex-biased mortality hypothesis”); (2) offspring born in the front portion of the national park are more likely to be killed than are those born in the remote area, on the grounds that human-caused mortality occurs near human settlements in most situations (“birthplace hypothesis”); (3) male-biased mortality, if any, is more evident in the remote area than in the front portion of the park on the grounds that females in the remote area are less likely to go outside the national park (“sex × birthplace hypothesis”); (4) offspring born in the front portion of the national park are likely to be killed at younger ages than are those born in the remote area, on the grounds that they become more vulnerable to human activity right after separation from their mother, even before they, especially males, initiate natal dispersal (“age × birthplace hypothesis”); and (5) offspring born to highly habituated mothers are more likely to be killed than are those born to less-habituated mothers, due to unwary behaviors inherited from the mother (“maternal human habituation hypothesis”). To test these hypotheses, we focused on adult female bears and their offspring inhabiting western Shiretoko National Park, dividing them into the following three groups according to their home ranges and levels of habituation to humans: group 1, females in the front portion of the national park with a moderate level of habituation; group 2, females at a medium distance from the front area with a low level of habituation; and group 3, females in the remote area with a very high level of habituation. We compared the likelihood of human-caused mortality of offspring by performing a long-term field study and genetic analysis.

## Methods

### Study area and animals

This study was conducted on the Shiretoko Peninsula (43°50′–44°20′ N, 144°45′–145°20′ E; Fig. 1), eastern Hokkaido, Japan. We conducted a monitoring survey of adult female bears inhabiting a region extending from the Horobetsu-Iwaobetsu area (44°5′ N, 145°1′ E; Fig. 1) to the Rusha area (44°11′ N, 145°11′ E), and divided the bears into three groups according to their home ranges and levels of human habituation. The home ranges were determined by the core areas revealed by radiotracking data for captured bears, and by the locations of DNA sampling (collection of hair, feces, and skin samples) for the remaining bears. The level of human habituation was determined based on the bears’ behavioral responses to humans, as follows: very high, no aversive reaction when approached by humans within 20 m; high, no aversive reaction to humans > 20 m away who move to within 20 m; moderate, movement away when approached within 50 m; and low, rare appearance before humans and rapid movement away upon awareness of humans. For bears inhabiting the area patrolled daily by park managers (i.e., the area excluding the Rusha area, as described below), this classification was done by two park managers with long experience with those bears from 1997 to 2018, based on patrol records that described the response of the bears when they approached to chase the bears away. We reviewed multiple randomly selected records for each bear and determined the habituation level based on the response most frequently observed. Alternatively, for bears inhabiting the Rusha area, outside the area patrolled for management purposes, two persons (one in common with the former case) determined the level, based on the response when they approached the bears multiple times during the long-term survey conducted between 2006 and 2018 (detailed in our previous reports^31^). All bears whose behavioral responses were not available, e.g., bears identified by genetic sampling that did not appear in front of humans, were categorized as low habituation level.

The first group comprised bears living around the front portion of the national park, including the Horobetsu-Iwaobetsu area, Shiretoko Five Lakes (44°7′ N, 145°5′ E), and the Utoro Plateau (44°3′ N, 144°59′ E), which is adjacent to the town of Utoro (44°4′ N, 144°59′ E; Fig. 1), a gateway community. Group 1 comprised 23 females, 14 of which were captured and released with ear tags for radiotracking in the Horobetsu-Iwaobetsu area between 1998 and 2013 and 9 of which were determined by genetic sampling to have been alive and present in this area from 1999 to 2018 [2 with distinctive characteristics (e.g., chest marking) were periodically identified in this area between 2013 and 2018]. This area is the main place where park visitors experience activities in nature. In the last two decades, bear sightings have been increasingly common in this area. When a bear appears near a road or development, park managers rush to the site and chase the bear away by shooting it with non-lethal rubber slugs and cracker shells. Thus, some bears are moderately habituated, but are not allowed to act freely during daylight hours. The second group comprised bears living between the Idashubetsu River (44°9′ N, 145°6′ E; Fig. 1) and the Rusha area, about 12.4–21.8 km from Utoro. Group 2 comprised 26 females, 4 of which were captured and released with ear tags for radiotracking in this area between 1993 and 2015 and 22 of which were determined by genetic sampling to have been alive and present in this area from 2000 to 2015 (13 were periodically identified in the area between 2013 and 2018). Kamuiwakka Falls (44°9′ N, 145°8′ E; Fig. 1) is the final tourist destination in this region; visitors are not allowed to pass through from Kamuiwakka Falls to the Rusha area. The third group comprised human-habituated bears living around the Rusha area, a special wildlife protection area with no public access without permission, and no human residence except for one fishermen’s settlement. Because the fishermen have not excluded bears from the settlement area in the last few decades, the bears have become habituated to the existence of humans and ignore people, which enables direct observation at close range (Fig. S1). Group 3 comprised 13 very highly habituated bears that had been visually identified and frequently observed in this area from the late 1990s to 2018^28,31–33^. Among them, six bears were captured and released with ear tags for radiotracking between 2013 and 2018^31^. Non- and less-habituated bears (low–high habituation levels) observed and sampled in the Rusha area were allocated to Group 2. All collared bears were captured live in accordance with the Guidelines for Animal Care and Use of Hokkaido University and all procedures were approved by the Animal Care and Use Committee of the Graduate School of Veterinary Medicine, Hokkaido University (Permit Number: 1152 and 15009, 17005, 18-0083). The protocols for capture received annual approval from the Ministry of the Environment, Japan, and the Hokkaido Government through research permit applications.

### Sample collection

During 1998–2018, we collected genetic samples from bears throughout the peninsula, including the towns of Shari, Rausu, and Shibetsu (Fig. 1), using multiple methods. Most samples were from bears that were dead due to nuisance control or hunting, or that were captured for research purposes. Blood, hair, liver, and/or muscle samples were obtained. Hunting is limited from October to January and is not allowed inside the national park. When a bear is killed, all hunters are required to report the reason why it was killed (*i*.*e*., nuisance control or hunting), date, location, sex, estimated age (based on body size), body mass, and size (if available), and to provide the genetic samples described above. Due to the strong relationship between park managers and hunters in the peninsula, poaching or hunting without a report are very unlikely to have occurred over the past two decades. In the current analysis, nuisance killing and hunting were included as human-caused mortality because, in most situations, hunting occurs near human settlements, especially around agricultural land, to mitigate the human–bear conflict in autumn, which makes it reasonable to consider that the likelihood of mortality by hunting is also influenced by the bears’ behavioral traits, including habituation level. We also obtained: (1) hair samples collected from hair traps, including fence traps and tree-rub traps placed in several locations (including the Horobetsu-Iwaobetsu and Rusha areas) during 2010–2018; (2) skin tissues collected by biopsy dart sampling during 2011–2018; and (3) fecal samples collected during 2009–2018. The sampling methods have been detailed in our previous reports^30–32,34^. For bears captured or killed between 1998 and 2018, age was estimated by counting the dental cementum annuli^35^.

### Human-caused mortality of the offspring of adult females

To determine how often offspring of adult females were killed due to human activities after separation from their mothers at 1.5–2.5 years of age^31^, the number of offspring (aged ≥ 1 year) produced by those females during the follow-up period must be known. However, we were unable to determine the exact number of offspring because the females, except for habituated bears in the Rusha area, were not monitored annually by direct observation. Therefore, the number of offspring produced by each female was estimated using the following formula: bear-years (follow-up years at ages ≥ 5 years) × reproductive rate (young born/year/reproductive adult female aged ≥ 5 years) × cub survival rate (from approximately 0.5 to 1.5 years of age). We assumed an equal sex ratio at birth. The latter two parameters were investigated in a long-term, individual-based monitoring survey in the Rusha area; values obtained were 0.74 and 0.63, respectively^31^. The bear-years were calculated from the year of first identification at ≥ 5 years of age (the earliest age of first reproduction in the Rusha area^31^) to the year of last identification. The initiation year was determined based on: (1) direct observation of a bear whose birth year was known, (2) dental age estimation at the time of capture or death, or (3) reproductive history (i.e., the age at first identification with cubs was assumed to be ≥ 5 years). The latter parameter was determined by not only direct observation of a female with cubs, but also indirect evidence of birth experience based on parentage analysis (described below). For example, if a female was assigned to the mother of 3-year-old bears (estimated by dental examination) killed in 2012, the mother was assumed to be ≥ 5 years old in 2009. The completion year was determined based on: (1) the year of last observation or death or (2) the reproductive history, revealed by parentage analysis. In the latter case, for example, if a female was assigned to the mother of 3-year-old bears in 2012, we assumed that the female survived at least until separation of the offspring (i.e., 2010), considering that a cub is not likely to survive its first year without the mother. Because genetic analysis of killed bears was initiated in 1998, the earliest year for the start of bear-year calculation was set at 1997, considering that offspring born in 1997 were potentially killed at 1 year of age in 1998. The latest year for bear-year calculation was set at 2015, as most human-caused mortality of offspring occurs at 1–3 years of age; thus, dead and alive bears born in 2015 can be evaluated in 2016–2018. The number of offspring (aged ≥ 1 year) killed by humans after separation from the mother was calculated by parentage analysis. Bears killed with their mothers were not included in the calculation. The age of offspring was estimated by counting the dental cementum annuli, for bears without birth records based on direct observation. Offspring sex was confirmed by genetic methods (described below) and external characteristics.

### DNA extraction and genotyping

DNA was extracted using the DNeasy Blood & Tissue Mini Kit (Qiagen Inc., Tokyo, Japan) for blood and tissue samples, the DNA Extractor FM Kit (Wako, Osaka, Japan) or Isohair Easy (Nippon Gene, Inc., Tokyo, Japan) for hair samples, and the QIAamp DNA Stool Mini Kit (Qiagen Inc.) for feces samples, according to the manufacturers’ protocols. Twenty-one microsatellite markers and one sex marker, amelogenin, were analyzed by multiplex PCR assay under conditions described previously (primers are listed in^31^). Allele size was determined using an ABI PRISM 310 genetic analyzer (Life Technologies Japan Ltd.). In addition, a mitochondrial DNA haplotype analysis targeting the control region was conducted to select candidate mothers of each individual in the parentage analysis (for details, see^30^).

### Parentage analysis

The parentage analysis was performed using a likelihood-based approach with CERVUS version 3.0.7^36^. The simulation parameters were 10,000 cycles, 150 candidate mothers and fathers per offspring, 40% of candidate parents sampled, and 1% of loci mistyped^32^. In the first step of the CERVUS analysis, we assigned a parent pair. The confidence level was set at 80%, and no mismatching was allowed in a parents–offspring combination (i.e., mother–father–offspring trio). One mismatch was allowed in a parents–offspring combination obtained at a ≥ 95% confidence level when the same mother and father were selected as the most likely parents (≤ 1 mismatch per pair) in maternity and paternity assignment analyses, respectively. If a parent pair could not be assigned due to a low (< 80%) confidence level or the presence of ≥ 1 mismatching loci, we assigned maternity as a second step. The confidence level was set at 80%, and no mismatching was allowed in a mother–offspring combination.

### Statistical analysis

Statistical analyses were performed with IBM SPSS Advanced Statistics ver. 23 (IBM Corp., Chicago, IL, USA). Due to several limitations in the dataset (including the limited sample size, wide variations of bear-years among the subject mothers, and biased geographical distribution of human habituated mothers), it was inappropriate to apply an individual-based approach, e.g., generalized liner model, to examine how multiple factors, including sex, proximity of the birthplace to areas of human activity, age, and human habituation of the mother, affect the likelihood of human-caused death of offspring. Instead, we applied group-based comparisons to test each hypothesis described in the Introduction. For all but the fourth hypothesis (age × birthplace hypothesis), we used Fisher’s exact test. For example, the first hypothesis (“sex-biased mortality hypothesis”) was tested by comparing the frequency of offspring mortality between males and females. The correspondences between each hypothesis and comparison pair are shown in Table 3. The method of Holm^37^ was used to adjust the *P* values obtained from multiple comparisons. In our analysis, the potential number of offspring produced by targeted mothers was estimated by bear-years, so the likelihood of human-caused death might be affected by this estimate, i.e., relatively few bear-years (small denominator) potentially increase the morality rate, especially in bears whose birth-years were determined by the genetic identification of the offspring, and not by direct observation or dental examinations. Therefore, the number of bear-years was compared among groups by one-way analysis of variance (ANOVA), followed by the Tukey–Kramer test to examine these possibilities. For the fourth hypothesis (age × birthplace hypothesis), group differences in ages of death for the offspring were analyzed by one-way ANOVA, followed by the Tukey–Kramer test. The homogeneity of variances in the data for bear-years and for ages of death was verified with Levene’s test. All comparisons were considered significant at *P* < 0.05. All values are presented as means ± standard deviations.

## Results and discussion

Our long-term, continuous survey between 1998 and 2018 enabled us to genotype 896 bears from which samples were collected. Among them, a total of 62 adult females were targeted as mothers in the analysis. The number of females at each habituation level and number of bear-years in each group is shown in Table 1. The parentage analysis revealed that 257 bears (130 males and 127 females) sampled during 1998–2018 were produced by the above-listed 62 females. Among them, 108 independent bears (aged ≥ 1 year, including 15 bears ≥ 5 years old) born during 1997–2015 were killed by humans during 1998–2018. The number of young (≥ 1 year) potentially produced by the mothers was estimated based on the bear-years in each group using the formula described above (Table 2). The overall human-caused mortality rate of bears aged 1–4 years was estimated as 35% (93/264; Table 2). This percentage may be an overestimate because the first bear-year was determined not by direct observation or dental age estimation, but by the first identification of offspring, for some females. Similarly, bear-year counts for some females were terminated before 2015, the end year of the bear-year count, although these individuals were not confirmed to be dead. Those bears might have produced more offspring before or after the bear-year period. Few of those bears were allocated to groups 1 (8/23) and 3 (1/13), but 15 of the 26 bears in group 2 were such, suggesting that the human-caused mortality rate in group 2 was overestimated, although the number of bear-years did not differ significantly among the three groups (Table 1; *P* > 0.05 for Levene’s test; *F*_[2, 59]_ = 2.03, *P* > 0.05 for one-way ANOVA).

**Table 1:** Number of adult females, their human habituation levels, and numbers of bear-years.

	Group 1	Group 2	Group 3
Main area	Utro Platau–Idashubetsu	Idashubetsu–Rusha	Rusha
Proximity to town^#^ (km)	0–12.1^a^	8.6^b^–21.8^c^	8.6^b^–21.8^c^
No. of subject females	23	26	13
Human habituation level (low/moderate/high/very high)	Low–High (17/2/4/0)	Low–High (23/2/1/0)	Very high (0/0/0/13)
No. of bear-years (Mean ± SD)	238 (10.3 ± 5.1)	192 (7.4 ± 5.3)	132 (10.2 ± 6.5)
No. of females killed*	7	6	1

^#^Proximity to town was calculated, (a) between Utoro and Idashubetsu river, (b) between Rusha area and Rausu town, and (c) between Utoro and Rusha area.

*The number of subject adult females that were killed for management purposes during the study period.

**Table 2:** Number of offspring born to adult females in each group that were killed by humans at an early stage of life (1–4 years of age).

	Group 1		Group 2		Group 3		Total	
	Male	Female	Male	Female	Male	Female	Male	Female
No. young* killed by humans	30	21	17	3	22	0	69	24
Total number of young*^#^	56	56	45	45	31	31	132	132

*Independent bears (1–4 years of age).

^#^Total number of young was estimated using the formula specified in the method.

The overall human-caused mortality rate was significantly higher in males than in females (52% *vs.* 18%; *P* < 0.01; Tables 2 and 3), which supports our first hypothesis (i.e., sex-biased mortality hypothesis). This tendency is in accordance with observations made in previous studies conducted on the Shiretoko Peninsula^28^ and in most brown bear populations^27,38,39^, in which the subadult mortality rate is highest among all age–sex classes. The sex difference was significant in groups 2 and 3 (males *vs.* females: 38% *vs.* 7% for group 2; 71% *vs.* 0% for group 3; both *P* < 0.01), but not in group 1 (*P* > 0.05), which supports our third hypothesis (i.e., sex × birthplace hypothesis). This strongly suggests that the primary underlying factor is male-biased natal dispersal^40^. Indeed, the occurrence of human-caused mortality peaked at 3 years of age in groups 2 and 3 (Table 4), which is consistent with the onset of dispersal of male brown bears^30,40^. In addition to their high mobility, young males tend to use areas close to humans or settlements^22^ and to be more active during the day^41^, resulting in a high mortality rate due to management killing and hunting.

**Table 3:** Summary of the statistical analysis by Fisher's exact test used for data in Table 2.

Comparisons	*P* value	*P*-Ajusted*	Hypothesis tested^#^
Male (total) vs Female (total)	< 0.0001	< 0.0013	H1 (s)
Group 1 (total) vs Group 2 (total)	0.0006	0.0054	H2 (s)
Group 1 (total) vs Group 3 (total)	0.21	0.42	H2 (ns)/H5 (ns)
Group 2 (total) vs Group 3 (total)	0.10	0.6	H5 (ns)
Group 1 (male) vs Group 1 (female)	0.13	0.65	H3 (s)
Group 2 (male) vs Group 2 (female)	0.0007	0.0056	H3 (s)
Group 3 (male) vs Group 3 (female)	< 0.0001	< 0.0013	H3 (s)
Group 1 (male) vs Group 2 (male)	0.16	0.64	H3 (s)
Group 1 (male) vs Group 3 (male)	0.17	0.51	H3 (s)/H5 (ns)
Group 2 (male) vs Group 3 (male)	0.0055	0.039	H5 (s)
Group 1 (female) vs Group 2 (female)	0.0003	0.003	H3 (s)
Group 1 (female) vs Group 3 (female)	< 0.0001	< 0.0013	H3 (s) / H5 (ns)
Group 2 (female) vs Group 3 (female)	0.27	0.27	H5 (ns)

*P values were adjusted by Holm's method (Holm S, 1979).

^#^This column indicates which comparison tested which hypothesis. H1, hypothesis 1 (sex-biased mortality hypothesis), H2, hypothesis 2 (birthplace hypothesis), H3, hypothesis 3 (sex × birthplace hypothesis), H5, hypothesis 5 (maternal human habituation hypothesis). “s” and “ns” indicate that each hypothesis was supported or not supported, respectively.

**Table 4:** Number of independent offspring killed by humans in each age class.

	Group 1		Group 2		Group 3		Total	
	Male	Female	Male	Female	Male	Female	Male	Female
1 year	9	2	0	1	1	0	10	3
2 years	16	6	5	1	8	0	29	7
3 years	4	8	9	1	9	0	22	9
4 years	1	5	3	0	4	0	8	5
Mean age^#^	1.9 ± 0.8*	2.8 ± 0.9	2.9 ± 0.7	2.0 ± 1.0	2.7 ± 0.8	—	2.4 ± 0.9	2.7 ± 1.0

*Significant difference compared to females in group 1, males in group 2, and males in group 3 (*P* < 0.01; Tukey–Kramer test). Females in group 2 and 3 were not included in the analysis due to limited sample number.

Offspring born in the front portion of the national park were more likely to be killed than were those born in the remote area (group 1 *vs.* 2: 46% *vs.* 22%; *P* < 0.01; Table 2), which supports our second hypothesis (i.e., birthplace hypothesis). This tendency was evident in females (group 1 *vs.* 2: 38% *vs.* 7%; group 1 *vs.* 3: 38% *vs.* 0%; both *P* < 0.01), but not in males (group 1 *vs.* 2: 54% *vs.* 38%; group 1 *vs.* 3: 54% *vs.* 71%; both *P* > 0.05), which also supports our third hypothesis (i.e., sex × birthplace hypothesis). This phenomenon reflected the natal philopatric nature of females^34,42^, male-biased natal dispersal^30^, and sex differences in home ranges (*i.e.*, smaller in young females than young males^43^). As much as 46% (51/112) of the offspring born to females in group 1 were killed due to human–bear conflict, which would disturb recruitment of their offspring into the population, thereby greatly reducing their fitness. In addition, among the monitored bears, the percentage of adult females that were killed due to human–bear conflict tended to be higher in group 1 (30%; Table 1) than in group 3 (8%), although this tendency was not statistically supported, presumably due to the limited sample size. In group 1, male offspring were killed at younger ages than were females (Table 3; *P* > 0.05 for Levene’s test; *F*_[3, 86]_ = 8.02, *P* < 0.001 for one-way ANOVA; *P* < 0.01 for Tukey–Kramer test), although no significant sex difference in mortality was observed (males *vs.* females: 54% *vs.* 38%; *P* > 0.05). Similarly, male offspring in group 1 were killed earlier than those in groups 2 and 3 (*P* < 0.01; Table 4), which supports our fourth hypothesis (*i.e.*, age × birthplace hypothesis). These findings suggested that males in the front portion of the park are more likely to be killed before the onset of natal dispersal^30^. One possible factor is a sex difference in energy demand. As Mattson and Reid^44^ predicted, subadult males are considered to require more energy for physical development than do subadult females, and so they experience greater energy stress, especially immediately after separation from their mothers. In searching for human-derived foods (e.g., garbage and agricultural crops), males aged 1–2 years may be more likely to approach human developments and agricultural land.

In contrast to the mortality rate of female offspring, which decreased toward the tip of the peninsula, that of male offspring was lowest in group 2 (38%) and highest in group 3 (71%) (*P* < 0.05 for group 2 *vs.* 3; Table 2). In group 3, no case of human-caused death was recorded among female offspring, whereas more than 70% of male offspring were killed by humans at 1–4 years of age (*P* < 0.01 for males *vs.* females). Considering natural death at 1–4 years of age, the total mortality rate for group 3 males should be much higher. In addition, although the proximity to human residential areas was comparable between groups 2 and 3, the mortality rate of male offspring in group 3 was approximately twofold higher than that in group 2; the latter may have been overestimated, as discussed above. These findings support our fifth hypothesis (*i.e.*, maternal human habituation hypothesis) in the case of male offspring. To our knowledge, this report is the first to indicate that the level of maternal human habituation, as well as offspring birthplace, age, and sex, determines the likelihood of human-caused mortality of the offspring at early stages of life, not only in bears, but also in large carnivores. Whether bears born to highly habituated mothers also become highly habituated is unclear; however, offspring adoption of parental habituation to humans has been reported in coyotes (*Canis latrans*)^45^, another carnivorous species. Social learning passed from mothers to their offspring would be a primary determinant of the level of human habituation. Bears stay near their natal area during the period between separation from their mothers at 1–2 years of age and the onset of natal dispersal at 2–3 years of age, which would be another process that increases the habituation levels in males born in the Rusha area^31^. The Rusha area has two key factors that facilitate human habituation^46^: (1) a high bear density, with aggregation in autumn due to the availability of salmon in the three rivers in the area; and (2) a high frequency of benign encounters with people including fishermen, researchers, and tourists on yachts. Therefore, most offspring presumably become more or less tolerant of people. Due to their natal philopatric nature and small home ranges (< 30 km^2^^29^), female offspring in the Rusha area are less likely to cause conflict near human settlement. In contrast, subadult males travel 18.3 km on average and 59.8 km at maximum^30^; at the dispersal site, even if near a human settlement, they behave as in the Rusha area, which makes them vulnerable to management killing. In this way, in the remote area of Shiretoko National Park, maternal human habituation has a profound negative impact on the survival of male offspring, reducing the mothers’ fitness.

Another consideration that should be entertained as a cause of the high mortality rate of group 3 males is the tendency to disperse to the east (i.e., to the town of Rausu; Fig. S1). Among the three research areas, the Rusha area is farthest (21.8 km) from the western park entrance, but relatively close (8.6 km) to the eastern side of the peninsula. The Shiretoko Mountain Range, reaching 1500 m in height, runs down the central axis of the peninsula, which may not act as a geographical barrier for males, but restricts the dispersal behavior of females. On the western side, management killing is concentrated around the town of Utoro, where human residences are concentrated, and in agricultural fields located near Utoro or the base of the peninsula (Fig. S2). The town of Rausu contains a fishermen’s base camp and human residences, but fewer agricultural farms, which are dotted from near the tip to the base of the peninsula, and intrusion into the human residential area is the main cause of management killing (Table S1). Habituated bears born in the Rusha area have little fear of humans and probably range near Rausu, even without utilizing accessible human-related foods. Because public complaints are often based on fear, rather than actual damage^47^, the unwary behavior of these bears explains the increased mortality rate in group 3.

When focused on the front portion of the park, the mortality rate of bears born to moderately or highly habituated mothers (9/23; 39%) did not differ from that of bears born to females with low habituation levels (42/88; 48%), although the number of moderately or highly habituated mothers was limited in group 1 (*n* = 6). This suggests that the enhancing effect of maternal human habituation on offspring mortality was masked in this area. One possible reason is that, even if the mother is not habituated, independent young may become habituated with the increasing frequency of benign encounters with people, especially tourists. Subadults tend to use the area close to human development^22^, which may accelerate human habituation. Another possibility is that, irrespective of the mother’s human habituation level, bears born in this area tend to become food conditioned due to the proximity to human residential areas and, in particular, to agricultural fields. Indeed, food conditioning was the leading cause of offspring mortality in group 1 (Table S1). In addition to the Utoro Plateau, agricultural fields of wheat, sugar beet, and dent corn are located on the southwestern side of the peninsula, and may act as an “attractive sink” where rich resources are available, but high numbers of anthropogenic deaths occur^48^.

The findings have some important implications for bear/wildlife management and conservation. First, the high human-caused mortality rate for males suggests that the recruitment of male offspring into the bear population is inhibited in the national park. Low recruitment of young males may not influence the productivity of the bear population, if the number of adult females is maintained. However, it may limit the turnover of reproductive males and allow a limited number of males to dominate reproductive opportunities, which would increase the occurrence of inbreeding and have a negative impact on genetic diversity^49^. Although inbreeding was not prominent in this population^33^, continued genetic monitoring is important. Second, even in the remote, special protection area of the national park (i.e., Rusha area), maternal human habituation enhances the likelihood of human–bear conflict. However, attempts to reduce habituation, *e.g.*, by aversive conditioning^50^, are not considered to be practical, not only because their efficacy is unclear^44^, but also because local communities, especially people organizing bear-viewing tour, would strongly oppose to it. Thus, effective countermeasures, although limited, such as the reduction of intrusion into human residential areas by electric fencing, are needed. Third, in the front portion of the national park, proximity to human settlement rather than maternal human habituation would be a more important factor that enhance the likelihood of human–wildlife conflict via food conditioning, which results in human-caused death outside the park. Due to the increasing number of bears in sightseeing areas over the last decade or so, park managers have been driving bears back to the forest by aversive conditioning. This approach might be effective for the prevention of dangerous encounters between people and bears, but might not be so effective to reduce human–bear conflict in human settlements near the front of the park. Rather, the prevention of food conditioning outside the park is thought to be easier and more effective.

## Conclusions

Our findings show that males born to habituated females in the remote area are likely to come into conflict with humans living around the national park, resulting in a high rate of mortality for management purposes. The Rusha area provides unique opportunities for park visitors to see wild bears and has come to symbolize the rich nature of the Shiretoko World Heritage Site. At a glance, it appears to be a bear sanctuary; however, the findings reveal that bears are not immune to human impacts in this narrow national park. The findings also suggest a negative effect of human habituation on the mother’s fitness in terms of reproductive success in Shiretoko National Park. However, there may still be a positive effect on it in terms of their own survival and that of the offspring after they mature, *e*.*g*., by increasing the opportunity to gain food resources^3,4,16^. Therefore, it is too early to state how natural selection shapes the proportion of habituated bears in the long run in this and other bear populations.

In conclusion, interactions among maternal human habituation, birthplace, age, and sex determine the likelihood of human-caused mortality of brown bears at an early stage of life. This was achieved only with long-term data, highlighting the importance of long-term, continuous surveys for wildlife studies. Human habituation of large carnivores is a key issue of growing concern to people living not only in rural, but also in urban, areas^51^. The current findings will contribute to the development of appropriate conservation and management strategies for large carnivores.

## Supplementary information

Supplementary Information.

## Data Availability

The data will be made available upon reasonable request.
