# Supplementary material for: Maternal human habituation enhances sons’ risk of human-caused mortality in a large carnivore, brown bears
Source: Sci Rep. 2020 Oct 5;10:16498. doi: 10.1038/s41598-020-73057-5 (PMC7536428; doi:10.1038/s41598-020-73057-5)
Supplement: Supplementary file 1 — Supplementary Information. [file 41598_2020_73057_MOESM1_ESM.docx]

**Supplementary Material**

**Title**

**Maternal human habituation enhances sons’ risk of human-caused mortality in a large carnivore, brown bears.**

Michito Shimozuru^1^*, Yuri Shirane^1^, Masami Yamanaka^2^, Masanao Nakanishi^2^, Tsuyoshi Ishinazaka^2^, Shinsuke Kasai^2^, Takane Nose^2^, Masataka Shirayanagi^2^, Mina Jimbo^1^, Hifumi Tsuruga^3^, Tsutomu Mano^3^, and Toshio Tsubota^1^

^1^ Laboratory of Wildlife Biology and Medicine, Faculty of Veterinary Medicine, Hokkaido University, Kita 18 Nishi 9, Kita-ku, Sapporo, Hokkaido 060-0818, Japan

^2^ Shiretoko Nature Foundation, 531 Iwaubetsu, Shari, Hokkaido 099-4356, Japan

^3^ Hokkaido Research Organization, Kita 19 Nishi 11, Kita-ku, Sapporo, Hokkaido 060-0819, Japan

**Table S1.** Causes of mortality of independent offspring (1–4 years of age). The causes of human-caused death were divided into five categories: 1) food conditioning [obsession with foods of human origin (*e.g.*, garbage, agricultural crops, fishery products)], 2) intrusion into human residential areas, 3) dangerous actions (*e.g.*, attack on a vehicle), 4) hunting, and 5) accidents (*e.g.*, traffic accidents).

|  | Group 1 | | Group 2 | | Group 3 | | Total | |
| --- | --- | --- | --- | --- | --- | --- | --- | --- |
|  | Male | Female | Male | Female | Male | Female | Male | Female |
| Food conditioning | 13 | 5 | 7 | 1 | 1 | 0 | 21 | 6 |
| Intrusion into human  residential areas | 11 | 9 | 10 | 1 | 17 | 0 | 38 | 10 |
| Dangerous actions | 3 | 3 | 0 | 0 | 2 | 0 | 5 | 3 |
| Hunting | 3 | 4 | 0 | 0 | 0 | 0 | 3 | 4 |
| Accidents | 0 | 0 | 0 | 1 | 2 | 0 | 2 | 1 |


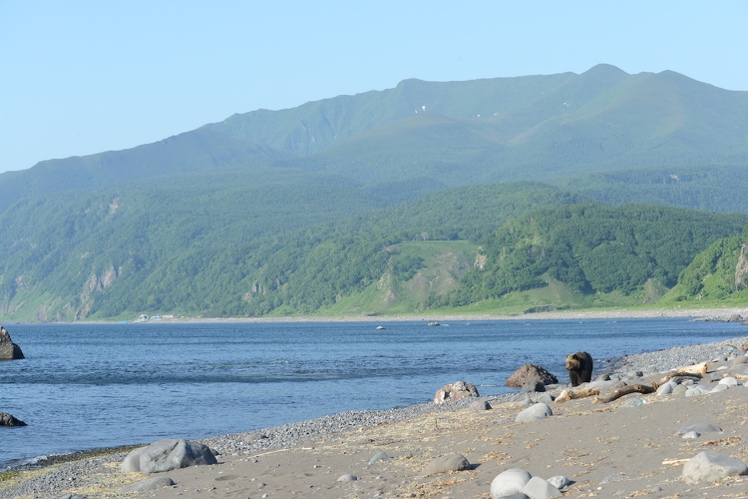


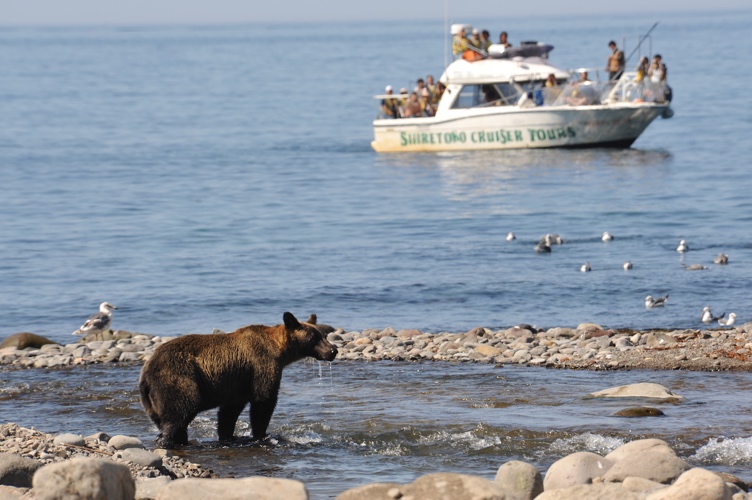


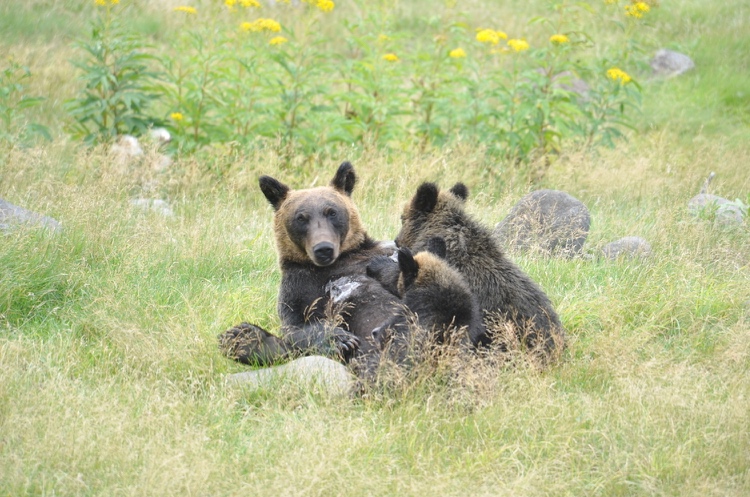


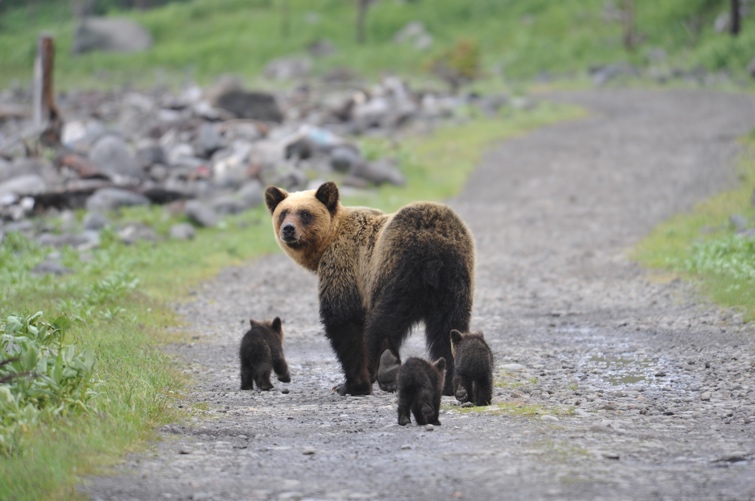


**Fig. S1.** Habituated female bears inhabiting the Rusha area.


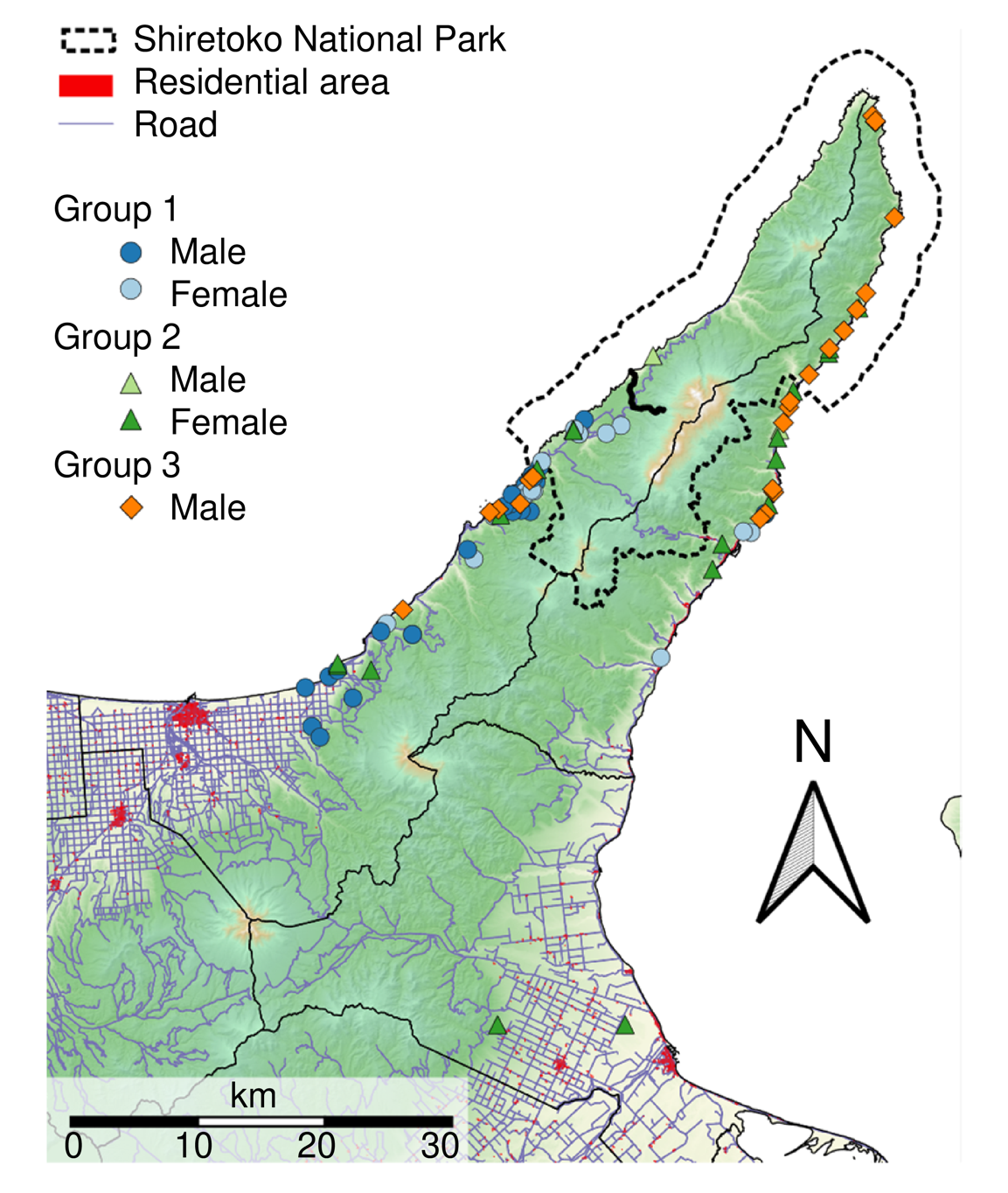
**Fig. S2.** Locations where offspring (aged 1–4 years) were killed by humans during 1998–2018. The dotted black line indicates the Shiretoko National Park. Males born to the mothers in Group 3 (orange rhombuses) tended to be killed at east side of the peninsula. This map was created using QGIS version 2.16 (QGIS Development Team, 2017. QGIS Geographic Information System. Open Source Geospatial Foundation Project. <http://qgis.osgeo.org>) and edited by M. Shimozuru. The base-map image, contour lines, topographic features are based on the National Land Numerical Information published by National Spatial Planning and Regional Policy Bureau, Ministry of Land, Infrastructure, Transport, and Tourism of Japan (available from <http://nlftp.mlit.go.jp/ksj/index.html>, accessed 7 December 2017).
